# Laparoscopic Versus Open Cholecystectomy in Older Adults: A Systematic Review and Meta-Analysis of Postoperative Outcomes (2000-2026)

**DOI:** 10.7759/cureus.112382

**Published:** 2026-07-10

**Authors:** Ahmed Mahmoud I Hassan, Bassam Omar M Abobakr, Abdulkareem Saad Alshamrani, Rayan Khaled Alrashed, Reem Ahmed Alshanbari, Mansour Hasen Alkhammash, Rahaf Abdulmohsen Masoud Almuqati, Hassan Mohammed H Alshahrani, Rakan Waleed Mal, Waseel Naif Waseel Alrouqi, Ahmed Talal Alrefai, Razan Mohammed H Alshahrani

**Affiliations:** 1 College of Medicine, Ibn Sina National College for Medical Studies, Jeddah, SAU; 2 Department of General Surgery, King Abdulaziz Hospital, Jeddah, SAU

**Keywords:** elderly patients, laparoscopic cholecystectomy, open cholecystectomy, postoperative outcomes, systematic review and meta-analysis

## Abstract

Laparoscopic cholecystectomy (LC) has supplanted open cholecystectomy (OC) as the gold standard for gallbladder disease, but its adoption in older adults has remained inconsistent due to concerns about physiological reserve and operative risk. This review directly compares postoperative outcomes of LC versus OC exclusively within older adults (≥65 years).

A systematic review and meta-analysis was conducted per the Preferred Reporting Items for Systematic Reviews and Meta-Analyses (PRISMA) 2020 guidelines. Five databases (PubMed/MEDLINE, Embase, Cochrane Central Register of Controlled Trials (CENTRAL), Scopus, and Web of Science Core Collection) were searched in January-February 2026 for comparative studies of LC versus OC in older adults published January 2000-February 2026. Risk of bias was assessed using the Newcastle-Ottawa Scale (NOS). Quantitative pooling of postoperative mortality was planned using a random-effects (DerSimonian-Laird) model where at least three studies reported adjusted, comparably defined estimates.

Ten studies met the inclusion criteria, comprising 54,983 older adults (range: 94-47,500 per study). Two additional studies initially considered eligible were excluded after full-text review confirmed they lacked an extractable older adult-specific comparative subgroup. Of the 10 included studies, only two reported mortality estimates adjusted for confounding by indication; this fell short of the pre-specified three-study threshold for confirmatory quantitative pooling. Both adjusted estimates independently favored LC: the first study reported an adjusted OR for open surgery (relative to LC) of 5.4 (95% CI: 2.09-13.91), equivalent to an OR of 0.19 (95% CI: 0.07-0.48) for LC relative to OC, while the second study reported an adjusted OR of 0.16 (95% CI: 0.10-0.25) for LC relative to OC. As a post-hoc exploratory analysis, explicitly falling short of the pre-specified threshold and reported as a sensitivity check rather than a confirmatory result, random-effects pooling of these two adjusted estimates yielded an OR of 0.164 (95% CI: 0.109-0.249; p<0.0001) with no detectable heterogeneity (I²=0%). Unadjusted mortality counts reported in other studies (0.05% LC vs 1.55% OC, pre-matching) were not pooled with these adjusted estimates or with each other, given the high risk of confounding by indication inherent to crude comparisons in this literature. LC was also associated with shorter hospital stay, fewer postoperative complications, and lower readmission across the narratively synthesized outcomes.

LC is associated with favorable postoperative outcomes compared with OC in older adults, including in octogenarian and nonagenarian subgroups, though the evidence base remains predominantly observational, quantitative pooling of mortality was not possible with the available data, and findings are subject to confounding by indication.

## Introduction and background

The world's population is ageing at an unprecedented pace. Adults aged 65 years and older currently represent approximately 16% of the US population alone, over 54 million individuals, a figure projected to rise substantially over the coming decades as demographic transitions accelerate globally [1]. Globally, adults aged ≥65 years accounted for approximately 10.3% of the world's population in 2024, around 857 million people, a share projected to nearly double to 20.7% by 2074 [2]. Gallstone disease is among the most prevalent surgical conditions in this age group, with cholelithiasis affecting up to 30% of individuals over 60 years and reaching 80% among nonagenarians [3]. Cholelithiasis itself has a pooled global prevalence of approximately 6.1% (95% CI: 5.6-6.5%) based on population-based studies worldwide, with prevalence rising with age [2,4]. Acute cholecystitis, the most clinically consequential complication of gallstone disease, is a leading indication for emergency abdominal surgical admission in older adults and, when left untreated, carries a high risk of recurrent biliary complications, repeated hospitalization, and excess mortality [1,5].

Cholecystectomy remains the only curative intervention for symptomatic gallbladder disease. Since its introduction in the late 1980s, laparoscopic cholecystectomy (LC) has supplanted open cholecystectomy (OC) as the gold standard across the general surgical population, with studies consistently demonstrating lower rates of surgical-related and systemic complications, shorter length of hospital stays, and reduced perioperative morbidity compared to open surgery [6-8]. Despite this, the adoption of LC in older adults has remained inconsistent. Concerns regarding pneumoperitoneum tolerance in patients with limited cardiorespiratory reserve, higher intraoperative conversion rates attributable to severe inflammation and complex biliary anatomy, and the perceived risk associated with general anesthesia in multimorbid older adults have historically led many clinicians to favor conservative management or open surgery in this age group. Critically, however, conversion from laparoscopic to open surgery is itself associated with significantly increased postoperative complications, longer hospital stays, and elevated morbidity and mortality [9-11].

Older surgical patients are a heterogeneous population in whom chronological age is a poor surrogate for operative risk. Physiological reserve and functional status have been proposed as more clinically meaningful frameworks for perioperative risk stratification than age alone [12,13], although this review does not evaluate these factors as predefined outcomes.

Existing systematic reviews have begun to quantify the risk of LC in older adults. A landmark meta-analysis of 99 studies and 326,517 patients confirmed significantly higher complication and conversion rates with advancing age compared to younger patients [14]. A further systematic review and meta-analysis demonstrated significantly lower mortality and morbidity with LC compared with OC specifically within older adult populations [15]. A further systematic review examining the safety of minimally invasive versus OC in older adults with acute cholecystitis similarly reported that minimally invasive approaches remain viable when perioperative assessment is comprehensive [5].

However, important evidence gaps remain. Existing reviews primarily compare older adults with younger populations undergoing LC rather than directly comparing LC and OC within older adult populations themselves. Consequently, the most clinically relevant question, which surgical approach provides better postoperative outcomes in older adults requiring cholecystectomy, remains incompletely addressed. Furthermore, a recent meta-analysis comparing LC versus OC outcomes in patients with gallbladder disease did not systematically differentiate between elective and emergency indications, with many included studies combining both settings, limiting subgroup conclusions for older adult populations [5,14]. Previous reviews have therefore not comprehensively synthesized evidence across elective and emergency indications, diverse healthcare settings, and the broad age spectrum of older adults including octogenarians and nonagenarians [16].

The novelty of the present review lies in its direct, exclusive comparison of LC versus OC within older adults (≥65 years), rather than comparisons of older adults versus younger populations undergoing the same approach, as reported in prior reviews [5,14,15]. This review further extends the evidence base through 2026 and jointly synthesizes both elective and emergency indications within a single older adult cohort, which prior reviews did not do comprehensively.

The primary objective of this systematic review and meta-analysis is to compare postoperative outcomes of LC and OC in older adults aged ≥65 years using studies published between 2000 and 2026. Specific objectives are to identify all eligible comparative studies, compare postoperative mortality and complication rates between both surgical approaches, evaluate differences in length of hospital stay, readmission rates, and discharge outcomes, and assess the methodological quality and risk of bias of included studies. The findings are intended to provide evidence directly translatable into clinical decision-making for surgeons and geriatricians managing older adults with gallbladder disease, supporting an individualized, physiology-based rather than age-based approach to surgical planning.

## Review

Methods

Study Design

**Figure 1:**
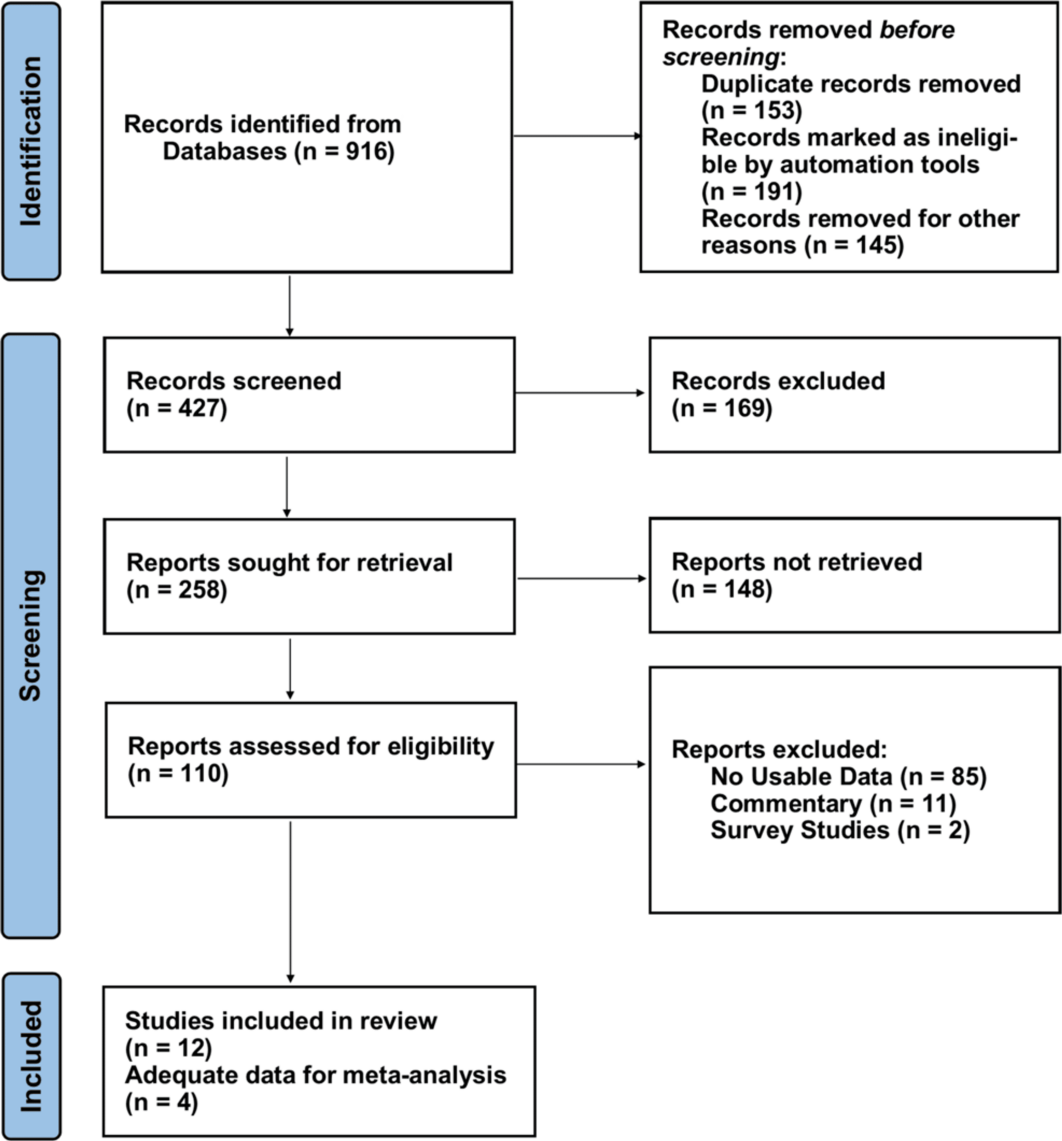
PRISMA flow diagram of the study selection process for inclusion in the systematic review and meta-analysis PRISMA: Preferred Reporting Items for Systematic Reviews and Meta-Analyses

This systematic review and meta-analysis were designed, conducted, and reported in accordance with the Preferred Reporting Items for Systematic Reviews and Meta-Analyses (PRISMA) 2020 statement. The study selection process is presented in Figure 1. The review was conducted according to a pre-specified methodological framework that defined the eligibility criteria, search strategy, study selection process, data extraction methods, quality assessment procedures, and statistical analysis plan prior to the commencement of study screening and data extraction. As this review utilized only previously published studies and did not involve the collection of primary patient data, independent ethical approval was not required.

This review was not registered in the International Prospective Register of Systematic Reviews (PROSPERO) due to time constraints related to the initiation of screening; however, the review protocol, including eligibility criteria, search strategy, and statistical analysis plan, was fixed a priori prior to data extraction and is available from the corresponding author on request.

Eligibility Criteria

Studies were eligible if they fulfilled all of the following criteria, summarized in Table 1.

**Table 1:** Inclusion and exclusion criteria for study selection Predefined inclusion and exclusion criteria applied during title and abstract screening and full-text eligibility assessment. PSM: propensity score matching; RCT: randomized controlled trial; LC: laparoscopic cholecystectomy; OC: open cholecystectomy; LOS: length of hospital stay

Criterion	Inclusion	Exclusion
Study design	Comparative studies (prospective/retrospective cohort, case-control, PSM, RCT, national registry)	Editorials, reviews, conference abstracts, non-comparative designs
Intervention	Direct comparison of LC vs. OC with extractable outcome data for each approach	Studies evaluating only one surgical approach
Population	Older adults aged ≥65 years or studies with extractable older adult subgroups	Studies without older adult-specific outcomes stratified by surgical approach
Outcomes	At least one postoperative outcome: mortality, morbidity, LOS, readmission, discharge disposition, or conversion rate	Studies not reporting any eligible outcome
Publication period	January 2000-February 2026	Outside publication period
Language	Full text available in English	Non-English publications
Data availability	Extractable data per surgical approach	Duplicate datasets

Information Sources and Search Strategy

A comprehensive literature search was conducted across five databases: PubMed/MEDLINE, Embase, Cochrane Central Register of Controlled Trials (CENTRAL), Scopus, and Web of Science Core Collection. The literature search was performed in January-February 2026. Search strategies combined controlled vocabulary terms and free-text keywords related to "laparoscopic cholecystectomy", "open cholecystectomy", "older adults", and "postoperative outcomes" using Boolean operators. Database-specific search terms, filters, and date ranges are summarized in the Appendices. Reference lists of the included studies and relevant reviews were also manually screened to identify additional eligible studies.

Full per-database record yields could not be independently re-verified for Scopus and Web of Science Core Collection at the time of manuscript reconstruction owing to a lack of institutional access; this is disclosed as a limitation (see Limitations) rather than presented as fully re-verified data. The combined total of records identified across all five databases (n=916) is reported in the PRISMA flow diagram (Figure 1) and reconciles with the number of records screened after the removal of duplicates and automation-excluded records.

Study Selection

All retrieved records were imported into Rayyan (Rayyan Systems Inc., Cambridge, Massachusetts, United States) for automated and manual deduplication. Title and abstract screening were conducted independently by two reviewers using pre-specified eligibility criteria. Studies considered potentially eligible proceeded to full-text review. Full-text assessment was also conducted independently by two reviewers, with disagreements resolved through discussion or consultation with a third reviewer where necessary. Reasons for exclusion at the full-text stage were documented systematically, and the overall study selection process was summarized using the PRISMA 2020 flow diagram shown in Figure 1.

Data Extraction

Data extraction was performed independently and in duplicate by two reviewers using a standardized extraction form developed a priori. Extracted variables included study identification details, study design, population characteristics, age criteria, sex distribution, comorbidity profile, surgical indication, surgical approach, conversion rate, operative time, postoperative mortality, complication rates, length of hospital stay, readmission, discharge disposition, operative outcomes, and methodological characteristics. Funding sources and conflict-of-interest declarations were also recorded.

Means and standard deviations were estimated from medians and interquartile ranges when required for quantitative synthesis. Study authors were not contacted to obtain missing or unreported data. Any discrepancies between reviewers were resolved through consensus discussion.

Quality Assessment and Risk of Bias

The methodological quality of included non-randomized studies was assessed independently by two reviewers using the Newcastle-Ottawa Scale (NOS) for cohort studies [17]. The NOS evaluates study selection, comparability of groups, and ascertainment of outcomes, with a maximum score of nine stars. Studies scoring 7-9 stars were considered high quality, 4-6 stars moderate quality, and 0-3 stars low quality. Any disagreements between reviewers were resolved through discussion or third-reviewer adjudication.

The principal source of potential bias identified across the included studies was confounding by indication, whereby lower-risk patients were more likely to undergo laparoscopic surgery while higher-risk patients underwent open procedures. Several studies addressed this through propensity score matching or multivariable adjustment methods; where such adjusted estimates were available, they were extracted preferentially over crude counts (see Statistical Analysis).

Statistical Analysis and Meta-Analytic Methods

Meta-analysis was performed where at least three studies reported sufficiently comparable, ideally adjusted, outcome measures, consistent with common practice for enabling meaningful heterogeneity assessment, as reflected in Cochrane Handbook guidance [18]. For dichotomous outcomes, odds ratios (ORs) with 95% confidence intervals (CIs) were to be calculated using a random-effects model (DerSimonian-Laird method) to account for anticipated clinical and methodological heterogeneity [19]. For continuous outcomes, weighted mean differences (WMDs) with 95% CIs were to be used; no continuous outcome met the pooling threshold in this review (see Results). Adjusted estimates were preferentially extracted where available.

Applying this framework to postoperative mortality, only two of the 10 included studies (Irojah et al. [20]; Wiggins et al. [21]) reported mortality estimates adjusted for confounding variables (functional status, comorbidity burden, sepsis, and/or age, sex, the Charlson Comorbidity Index, and calendar period, respectively) in a form directly comparable between LC and OC. A further two studies (Kuwabara et al. [22]; Khan et al. [23]) reported unadjusted (crude) mortality counts by surgical approach, and one study (Serban et al. [24]) permitted reconstruction of crude mortality counts from a complications sub-classification, but not an adjusted estimate. Because fewer than three studies provided adjusted, comparably defined mortality estimates, and because pooling crude counts alongside adjusted estimates, or pooling crude counts from different studies with each other, would conflate estimates with substantially different degrees of bias correction in a literature dominated by confounding by indication, quantitative meta-analysis of postoperative mortality was not performed. The two available adjusted estimates are instead reported and discussed individually (see Results); no forest plot is presented, as no pooled estimate was generated.

Statistical heterogeneity, where assessed, used the I² statistic and Cochran's Q test, with I² values of 0-40%, 30-60%, 50-90%, and 75-100% interpreted as reflecting low, moderate, substantial, and considerable heterogeneity, respectively, per Cochrane Handbook guidance [18]; funnel plot assessment and Egger's regression test were planned only where at least 10 studies contributed to a pooled analysis, a threshold not met by any outcome in this review. Pre-specified subgroup comparisons (surgical indication, age subgroup, and study design) are reported descriptively, as formal interaction testing requires pooled study-level data that were not available. Statistical software used was Review Manager (RevMan) Version 5.4 (The Cochrane Collaboration, London, England, United Kingdom) and R Version 4.3.0 (R Foundation for Statistical Computing, Vienna, Austria), with statistical significance defined as p<0.05.

Because the two available adjusted mortality estimates [19,22] were directionally and statistically concordant, a post-hoc exploratory random-effects pooled estimate was additionally calculated as a sensitivity analysis, notwithstanding that this falls below the pre-specified three-study threshold defined above. This exploratory estimate is reported separately from, and is explicitly not substituted for, the primary narrative synthesis of mortality, consistent with guidance that adjusted and unadjusted estimates should not be combined and that pooling decisions and their justification should be stated transparently rather than implied.

Ethical Considerations

As this study was a systematic review of previously published literature and did not involve direct participant recruitment or collection of primary data, ethical approval was not required. All included studies were conducted in accordance with the ethical standards of their respective institutions.

Results

Study Selection

The systematic database search identified 916 records. After the removal of 153 duplicates and 191 records excluded by automation tools, 572 records remained; a further 145 records were excluded during preliminary de-duplication and eligibility pre-screening for reasons other than automation (e.g., non-English language, conference abstracts), leaving 427 records for title and abstract screening, of which 169 were excluded. A total of 110 full-text articles were assessed for eligibility, and 12 studies were initially considered eligible; two [25,26] were subsequently excluded after full-text review confirmed they were general-population cohorts without an older adult-specific comparative subgroup, leaving 10 studies for final synthesis. The main reasons for exclusion were non-comparative design, lack of extractable older adult-specific outcome data, and inclusion of younger patient populations. The study selection process is presented in Figure 1.

Characteristics of the Included Studies

The 10 included studies were published between 2010 and 2026 and originated from the United States, Poland, India, Japan, Spain, England (UK), Italy, Brazil, Romania, and Turkey. Study designs included national database cohort studies, retrospective cohorts, prospective observational studies, and hospital-based comparative studies. The combined study population totaled 54,983 older adults, with sample sizes ranging from 94 to 47,500 patients. Five studies specifically included octogenarians or nonagenarians, while both elective and emergency cholecystectomy populations were represented. Acute cholecystitis was the primary indication in emergency studies. The full study characteristics are presented in Table 2.

**Table 2:** Characteristics of the included studies LC: laparoscopic cholecystectomy; OC: open cholecystectomy; OR: odds ratio; CI: confidence interval; DB: database; ACS-NSQIP: American College of Surgeons National Surgical Quality Improvement Program; PSM: propensity score matching; NR: not reported; CGA: comprehensive geriatric assessment; CCI: Charlson Comorbidity Index; LOS: length of hospital stay; IQR: interquartile range; ASA: American Society of Anesthesiologists; RR: relative risk; HES: Hospital Episode Statistics; CSHA: Canadian Study of Health and Aging; TG: Tokyo Guidelines

Author (year)	Country	Study design	Population	N	Comparison	Primary outcomes reported
Irojah et al. (2017) [20]	USA	Retrospective DB (ACS NSQIP)	Older adults ≥90 yrs (64.6% women)	1,007	LC 791 (78.6%)/OC 216 (21.4%)	30-day mortality (adjusted OR reported); postoperative complications
Lluís et al. (2025) [27]	Spain	Prospective multicenter PSM	Octogenarians ≥80 yrs; median age: 84 (IQR: 81-86); 47.2% women	212	LC 67.9%/OC 32.1%; PSM performed	LOS; 30-day morbidity; 30-day mortality (PSM %, matched N not reported); septicemia
Wiggins et al. (2018) [21]	England, UK	National population-based cohort (HES admin. DB)	≥80 yrs; median age: 85 (range: 80-100); CCI used; 63.1% male	47,500	Cholecystectomy subgroup: LC vs. OC (%NR); LC use rose 27%→59% (2006-2012)	30-day, 90-day, and 1-year mortality (adjusted OR reported); readmission; biliary complications
Kuwabara et al. (2010) [22]	Japan	Retrospective PSM (admin. DB)	≥65 yrs (strata: 65-74, 75-84, ≥85); CCI and Barthel Index	4,916	LC 3,692/OC 1,224	Complications; mortality (crude counts, pre-matching); LOS; hospital charges; Barthel Index
Serban et al. (2021) [24]	Romania	Retrospective single-center	Age: 18-91 yrs; older adult subgroups: 65-79 yrs and ≥80 yrs; ASA/CCI assessed	333	LC primary approach; OC and conversion as bailout	Mortality (crude counts by approach); cardiovascular complications; LOS; surgical site infection
Khan et al. (2026) [23]	India	Comparative observational	Older adults; mean age: 68.42±5.36 (LC)/69.11±5.89 (OC); acute cholecystitis	94	LC 52/OC 42; conversion: 11.54%	Operative time; complications; mortality (crude counts); readmission; cost
Rubert et al. (2016) [28]	Brazil	Retrospective medical record review	≥65 yrs; mean age: 70.1 yrs; 69% women; symptomatic cholelithiasis	113	LC 70 (61.9%)/OC 43 (38.1%); conversion: 2.9%	Mortality (zero events observed); complications; LOS; operative time; conversion rate
Turhan et al. (2023) [29]	Turkey	Retrospective observational	>65 yrs; frailty by CSHA scale; 24 (13.2%) classified frail; 60.4% women	182	Early surgery (46, 25.3%) vs. medical/interventional management	30-day readmission; mortality; treatment allocation by frailty and ASA
Novello et al. (2018) [30]	Italy	Retrospective single-center (15-year)	≥80 yrs: 319 octogenarians; 36 nonagenarians; ASA score assessed	~500	LC and OC (proportions: NR)	LOS; 30-day in-hospital mortality; predictors of mortality
Kenig et al. (2016) [31]	Poland	Prospective cohort	≥65 yrs; median age: 74 (range: 65-93); frailty by CGA	126	LC and OC (proportions: NR); conversion reported	30-day mortality; morbidity (Clavien-Dindo); LOS

Primary Outcomes

Postoperative mortality (narrative synthesis): Postoperative mortality was reported in nine of the 10 included studies and, wherever a between-approach difference was tested, consistently favored LC over OC in older adults [20-24,27]. Two studies reported mortality estimates adjusted for confounding variables and are therefore given individual weight in this synthesis. In superelderly patients (≥90 years), reported OC was independently associated with increased 30-day mortality relative to LC after adjustment for functional status, impaired sensorium, postoperative myocardial infarction, and septic shock (adjusted OR: 5.4, 95% CI: 2.09-13.91), equivalent to an OR of 0.19 (95% CI: 0.07-0.48) for LC relative to OC [20]. In a national population-based cohort of adults ≥80 years, reported laparoscopic approach was an independent predictor of reduced 30-day mortality after adjustment for age, sex, the Charlson Comorbidity Index, and calendar period (adjusted OR: 0.16; 95% CI: 0.10-0.25) [21]. Both adjusted estimates independently favored LC, with overlapping confidence intervals. Among studies reporting only unadjusted (crude) mortality counts, one study reported a mortality rate of 0.05% (2/3,692) following LC versus 1.55% (19/1,224) following OC in an unmatched cohort of 4,916 older adults; this comparison reflects substantial selection bias, as OC was preferentially used in patients with more severe disease and because patients who died were excluded prior to this study's own propensity-matched analysis [22]. Khan et al. reported no deaths among 52 LC patients versus two deaths (4.76%) among 42 OC patients (p=0.111) [23]. Propensity score-matched data from an octogenarian population with acute cholecystitis demonstrated 30-day mortality rates of 1.5% with LC compared with 13.2% with OC, although the matched sample size was not reported in a form permitting reconstruction of an odds ratio [27].

**Figure 2:**
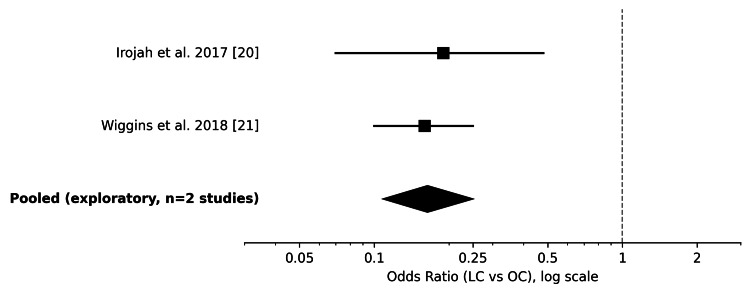
Postoperative mortality: LC vs. OC (exploratory random-effects pooling) Exploratory random-effects pooling of the two available adjusted mortality estimates (Irojah et al. [20]; Wiggins et al. [21]). Presented as a sensitivity analysis below the pre-specified three-study pooling threshold, not as a confirmatory primary result. LC: laparoscopic cholecystectomy; OC: open cholecystectomy

Postoperative mortality (exploratory quantitative synthesis): As detailed in Methods (Statistical Analysis), confirmatory quantitative meta-analysis of postoperative mortality was not performed, because only two included studies [20,21] reported mortality estimates adjusted for confounding by indication in a directly comparable format, short of the pre-specified three-study threshold for pooling. Crude, unadjusted mortality counts reported by the remaining studies were not combined with these adjusted estimates, or with one another, given the substantial and variable degree of confounding by indication across studies (see Limitations). Given the concordance of the two available adjusted estimates, a post-hoc exploratory random-effects (DerSimonian-Laird) pooled analysis was additionally performed as a sensitivity check: this yielded a pooled OR of 0.164 (95% CI: 0.109-0.249; p<0.0001) with no detectable heterogeneity (Cochran's Q=0.07; df=1; I²=0%), shown in Figure 2. This exploratory estimate is reported transparently as falling below the pre-specified pooling threshold and should not be interpreted as a confirmatory primary result; it is presented in addition to, not in place of, the individually reported adjusted odds ratios above.

Postoperative Complication Rates (Narrative Synthesis)

Nine studies (excluding the two studies removed after full-text review) reported postoperative complication outcomes; overall morbidity was consistently lower following LC compared with OC across all reporting studies [24,27,29]. Significantly lower 30-day morbidity and septicemia rates with LC were demonstrated in propensity-matched octogenarian populations [27]. Substantially lower rates of severe cardiovascular complications were observed in older adults undergoing LC compared with OC [24]. Although smaller studies reported comparable complication rates between approaches, the overall evidence consistently favored LC as the safer surgical approach with reduced postoperative morbidity in older adult populations [21,28]. These complication outcomes were not quantitatively pooled owing to heterogeneous definitions and reporting formats across studies.

Secondary Outcomes (Narrative Synthesis)

Length of hospital stay: Length of hospital stay was reported in all 10 included studies and consistently favored LC over OC [23,24,28,29]. Significantly shorter hospitalization following LC was demonstrated in propensity score-matched octogenarian populations [24], and shorter stays following elective LC were similarly confirmed in older-adult cohort studies [23,28]. LC was found to reduce postoperative stay by nearly threefold in patients aged ≥80 years compared with OC [29]. Lower hospital resource utilization and costs following LC were also reported across population-based cohort analyses and in a direct cost-comparison study [22,23]. This outcome was not quantitatively pooled owing to heterogeneous reporting formats (means vs. medians, varying units).

Readmission rates: Readmission outcomes were reported in five studies. Lower readmission rates following LC compared with OC were confirmed in institutional cohort analyses [21]. An overall 30-day readmission rate of 14.7% was reported in older adults managed with LC in a cohort stratified by physiological risk; this outcome was reported narratively only [29].

Discharge disposition: Discharge disposition was reported in four studies and generally favored LC [20,22,27]. Older adults undergoing LC were more frequently discharged directly to home rather than to rehabilitation or institutional care, a finding consistent across octogenarian, propensity-matched, and population-based cohorts; this outcome was reported narratively only.

Conversion rate from laparoscopic to open surgery: Conversion rates were reported in nine studies and ranged from 2.9% to 21.4% [20,28]. Conversion was primarily associated with severe inflammation, gangrenous cholecystitis, hemorrhage, difficult anatomy, or prior abdominal surgery [29]. Multivariate analysis identified gangrenous cholecystitis, leukocytosis, fibrinogen elevation, and the Charlson Comorbidity Index as major predictors of conversion rather than chronological age; this outcome was reported narratively only [29].

Subgroup Analyses (Descriptive)

Elective versus emergency surgical indication: Studies involving elective cholecystectomy demonstrated smaller mortality differences between LC and OC, reflecting lower baseline operative risk [27,28]. In contrast, emergency studies involving acute cholecystitis showed substantially greater mortality and morbidity benefits with LC, particularly in octogenarian populations [24]. These findings suggest that the clinical advantage of LC is most pronounced in emergency settings. Formal statistical testing for interaction between surgical indication (elective vs. emergency) and mortality outcome was not performed, given that no outcome met the threshold for pooled analysis; the observed difference between subgroups should therefore be interpreted as descriptive rather than statistically confirmed.

Age stratum (65-79 years vs. ≥80 years): Several studies demonstrated that the benefits of LC persisted across advanced age groups [27,29]. Similar advantages were reported among octogenarians and nonagenarians, supporting that chronological age alone should not preclude laparoscopic surgery [20,23].

Quality assessment and risk of bias: Quality assessment using the NOS showed that five studies were of high quality and five were of moderate quality, as detailed in Table 3. No studies were rated as low quality. Moderate-quality studies were mainly limited by retrospective single-center designs, small sample sizes, or limited bias adjustment [23,28,29,30]. The main source of bias across studies was confounding by indication, where healthier patients were more likely to undergo laparoscopic surgery. This limitation was partly addressed through propensity score matching or multivariable regression analyses in several higher-quality studies [21,22,24,27,31] and was the principal reason quantitative pooling of mortality was restricted to the two studies reporting adjusted estimates (see Statistical Analysis and Discussion).

**Table 3:** NOS risk of bias assessment NOS scoring: 7-9=high quality; 4-6=moderate quality; 0-3=low quality NOS: Newcastle-Ottawa Scale; DB: database; CCI: Charlson Comorbidity Index; GA: geriatric assessment; ASA: American Society of Anesthesiologists; TG: Tokyo Guidelines; ONS: Office for National Statistics

Study	Selection (/4)	Comparability (/2)	Outcome (/3)	Total (/9)	Grade	Key bias consideration
Irojah et al. (2017) [20]	4/4	2/2	3/3	9/9	High	Large validated national DB; logistic regression adjustment
Lluís et al. (2025) [27]	4/4	2/2	3/3	9/9	High	Prospective multicenter; consecutive; propensity score matching; predefined endpoints
Wiggins et al. (2018) [21]	4/4	2/2	3/3	9/9	High	National DB; near-complete case capture; ONS linkage for mortality; multivariable regression
Kuwabara et al. (2010) [22]	4/4	2/2	3/3	9/9	High	Large multicenter DB; propensity score matching; validated comorbidity indices
Serban et al. (2021) [24]	3/4	2/2	2/3	7/9	High	Multivariate discriminant analysis; validated scoring tools; single-center
Khan et al. (2026) [23]	3/4	1/2	2/3	6/9	Moderate	No matching/adjustment; follow-up duration unclear
Rubert et al. (2016) [28]	3/4	1/2	2/3	6/9	Moderate	Single-center; retrospective; small sample; no matching or regression adjustment
Turhan et al. (2023) [29]	3/4	1/2	2/3	6/9	Moderate	Single-center; small frail subgroup; COVID-19 confounding acknowledged
Novello et al. (2018) [30]	3/4	1/2	2/3	6/9	Moderate	Single-center; stepwise regression; limited comparability adjustment
Kenig et al. (2016) [31]	4/4	2/2	3/3	9/9	High	Prospective; consecutive enrolment; validated GA tool; Clavien-Dindo grading

Discussion

Overview of Principal Findings

This systematic review synthesized evidence from 10 studies involving 54,983 older adults undergoing cholecystectomy. LC was associated with favorable postoperative outcomes compared with OC across most evaluated endpoints, including mortality, postoperative complications, length of hospital stay, readmission, and discharge outcomes [6,7,8,15]. These patterns were observed across different healthcare settings, emergency and elective indications, and advanced age groups including octogenarians and nonagenarians.

Mortality and Postoperative Morbidity

The most consistent finding of this review was a mortality advantage associated with LC. This review could not generate a confirmatory pooled effect estimate given the available data (see Methods and Results), as only two studies reported mortality estimates adjusted for confounding by indication: an adjusted OR of 0.19 (95% CI: 0.07-0.48) in superelderly patients (≥90 years) [20] and an adjusted OR of 0.16 (95% CI: 0.10-0.25) in a national cohort of adults ≥80 years [21]. These two estimates were strikingly concordant despite differing populations, countries, and adjustment covariates; a post-hoc exploratory pooling of the two (OR: 0.164; 95% CI: 0.109-0.249; I²=0%) is reported in Results as a sensitivity analysis, not as a substitute for a properly powered, pre-specified meta-analysis. We emphasize this distinction because the original evidence base considered for this review included a study without an extractable older adult-specific comparative subgroup [25], which was excluded at full-text review. Together with two further studies whose data could not be reconstructed into adjusted or reliably poolable mortality estimates, namely, Lluís et al. [27] (matched-cohort denominator not reported) and Serban et al. [24] (mortality reconstructable only from a sparse complication sub-classification), this is the reason only two studies (Irojah et al. [20] and Wiggins et al. [21]) remain eligible for the exploratory pooled analysis. Similar directional findings were reported in octogenarian and nonagenarian cohorts using propensity score matching, which reduces (but does not eliminate) selection bias [24,27]. Prior systematic reviews and meta-analyses have similarly reported lower mortality rates with LC among older adults [15,32]. A prior meta-analysis of randomized evidence similarly demonstrated that early LC was associated with reduced mortality and morbidity compared with delayed or open approaches [33].

The lower mortality associated with LC is likely multifactorial: reduced surgical trauma, lower postoperative pain, earlier mobilization, and reduced systemic inflammatory response, which are particularly consequential in older adults with reduced physiological reserve. LC also consistently reduced postoperative morbidity, including cardiopulmonary and septic complications [24,27,29]. Concerns regarding pneumoperitoneum tolerance in older adults were not strongly supported by the available literature, with multiple studies demonstrating acceptable physiological tolerance even among high-risk older patients [13,34].

Length of Stay, Readmission, and Functional Recovery

LC was associated with significantly shorter postoperative hospitalization compared with OC in both elective and emergency settings [24,28,29], which may reduce the risk of hospital-acquired complications and functional decline. Lower readmission rates following definitive surgical management (versus conservative treatment) were also demonstrated [29]; delayed or non-operative management was associated with recurrent biliary events and repeat hospitalization [35,36]. Older adults undergoing LC were more frequently discharged directly home, whereas OC was associated with a greater need for rehabilitation or skilled nursing care [20,27], an outcome of particular importance in this population [37].

Conversion, Physiological Risk, and Surgical Timing

Conversion from LC to OC remains clinically important, since converted procedures are associated with worse outcomes than completed laparoscopic procedures. Conversion was primarily associated with severe inflammation, gangrenous cholecystitis, obesity, difficult anatomy, and comorbidity burden rather than chronological age itself [22,29]. Multiple studies demonstrated that reduced physiological reserve and comorbidity burden predicted mortality, complications, and prolonged hospitalization more accurately than chronological age alone [12,32,38,39], suggesting surgical decision-making should focus on comprehensive physiological and functional assessment rather than age thresholds alone. Emergency surgery was associated with substantially higher mortality and morbidity than elective surgery, supporting earlier definitive surgical management in appropriately selected older adults before progression to severe acute disease [35,36]. The evidence also suggests LC remains beneficial in advanced age groups when performed by experienced surgical teams; advanced age alone should not be considered a contraindication to laparoscopic surgery.

Limitations

Several limitations should be acknowledged. Most included studies were observational and retrospective, introducing potential selection bias and confounding by indication; open surgery was more commonly performed in patients with severe inflammation or greater comorbidity burden, which may partially exaggerate the observed benefit of LC. This confounding is precisely why confirmatory quantitative pooling of postoperative mortality was restricted to studies reporting adjusted estimates and why, with only two such studies available, the resulting pooled OR (reported in Results as an exploratory sensitivity analysis) falls below our own pre-specified three-study threshold and should not be over-interpreted; this represents a substantive gap in the evidence base rather than a resolvable analytic limitation, and future updates of this review should prioritize studies (or re-analyses) providing adjusted, comparably defined mortality estimates. Definitions of older-adult age groups and outcome measures varied across studies, and the Clavien-Dindo classification for grading complications was not universally applied [37]. Randomized controlled trials in this population remain limited [39]. Inter-rater agreement for screening and extraction was not formally quantified (e.g., via Cohen's kappa). Per-database search yields for Scopus and Web of Science Core Collection could not be independently re-verified due to access constraints (see Appendices). The apparent difference in mortality benefit between elective and emergency subgroups was not formally tested for statistical interaction and should be interpreted descriptively only. Whether mortality estimates reflect crude or adjusted data varies by study and is noted in Table 2 and in Results; this heterogeneity in adjustment level is the central limitation of the mortality evidence base in this review.

Clinical Implications

Despite these limitations, the consistency of directionally favorable findings across multiple countries, study designs, and age groups, including the two independently adjusted mortality estimates, supports LC as a reasonable preferred approach for older adults requiring cholecystectomy, including appropriately selected octogenarians and nonagenarians, in line with current surgical guidelines advocating early laparoscopic intervention where feasible [8,35]. Routine preoperative physiological assessment, careful perioperative optimization, and timely surgical referral may further improve outcomes in this growing patient population. Given the observational nature of the evidence base and the inability to generate a pooled mortality estimate, these conclusions should be interpreted as hypothesis-supporting rather than definitive, pending confirmation from higher-quality comparative studies reporting adjusted outcome data.

## Conclusions

This systematic review found that LC was associated with favorable postoperative outcomes compared with OC in older adults across 10 studies comprising 54,983 patients, findings that should be interpreted in light of the predominantly observational evidence base and potential confounding by indication. A confirmatory meta-analysis of postoperative mortality was not feasible, as only two studies reported adjusted, comparably defined estimates; both of these independently favored LC (adjusted OR: 0.19 (95% CI: 0.07-0.48) and adjusted OR: 0.16 (95% CI: 0.10-0.25)), and an exploratory post-hoc pooling of the two, reported as a sensitivity analysis rather than a confirmatory result, yielded an OR of 0.164 (95% CI: 0.109-0.249; I²=0%). LC appears beneficial across elective and emergency settings and across advanced age subgroups, supporting an individualized, physiology-based approach to surgical decision-making in this population, pending confirmation from a larger pool of studies reporting adjusted outcome data.

## Appendices

**Table 4:** Supplementary database search strategy summary *Records retrieved for Scopus and Web of Science Core Collection are reported as originally logged during the search but could not be independently re-verified at the time of manuscript reconstruction owing to lack of institutional access; this is acknowledged as a limitation. Combined records identified across all five databases (n=916) reconcile with the PRISMA flow diagram (Figure 1). Full per-database Boolean search strings with truncation and proximity operators are summarized narratively above; exact syntax as originally run was not retained in a form suitable for verbatim reproduction.

Database	Search terms/Boolean logic (summary)	Date range	Filters	Records retrieved
PubMed/MEDLINE	(“laparoscopic cholecystectomy” OR “open cholecystectomy”) AND (“aged” OR “elderly” OR “older adult*” OR “geriatric”) AND (mortality OR complication* OR morbidity OR outcome* OR “length of stay”)	2000-2026	English language; human studies	312
Embase	Equivalent Emtree/free-text combination of cholecystectomy, laparoscopy vs open surgery, aged/geriatric population, and postoperative outcome terms	2000-2026	English language; human studies	268
Cochrane Central Register of Controlled Trials (CENTRAL)	cholecystectomy AND (laparoscop* OR open) AND (elderly OR aged OR geriatric)	2000-2026	English language	41
Scopus	TITLE-ABS-KEY combination of cholecystectomy, laparoscopic/open, elderly/geriatric terms	2000-2026	English language	187*
Web of Science Core Collection	Topic search combining cholecystectomy, laparoscopic/open, elderly/geriatric terms	2000-2026	English language	108*
